# Key gene modules and hub genes associated with pyrethroid and organophosphate resistance in *Anopheles* mosquitoes: a systems biology approach

**DOI:** 10.1186/s12864-024-10572-z

**Published:** 2024-07-03

**Authors:** Cynthia Awuor Odhiambo, Dieunel Derilus, Lucy Mackenzie Impoinvil, Diana Omoke, Helga Saizonou, Stephen Okeyo, Nsa Dada, Nicola Mulder, Dorothy Nyamai, Steven Nyanjom, Audrey Lenhart, Luc S. Djogbénou, Eric Ochomo

**Affiliations:** 1 Department of Biochemistry, Jomo Kenyatta University of Agriculture and Technology, Nairobi, Kenya; 2 Kenya Medical Research Institute (KEMRI), Centre for Global Health Research (CGHR), Kisumu, Kenya; 3 Division of Parasitic Diseases and Malaria, Entomology Branch, Centers for Disease Control and Prevention (CDC), Atlanta, GA USA; 4 Tropical Infectious Diseases Research Center (TIDRC), University of Abomey-Calavi (UAC), Cotonou, Benin; 5 School of Life Sciences, Arizona State University, Tempe, AZ USA; 6 Human, Heredity, and Health in Africa H3A Bionet Network, Cape Town, South Africa; 7 Regional Institute of Public Health (IRSP), Ouidah, Benin; 8 Liverpool School of Tropical Medicine, Liverpool, UK

**Keywords:** Insecticide resistance, *Anopheles gambiae*, *Anopheles arabiensis*, Hub genes, Molecular markers

## Abstract

Indoor residual spraying (IRS) and insecticide-treated nets (ITNs) are the main methods used to control mosquito populations for malaria prevention. The efficacy of these strategies is threatened by the spread of insecticide resistance (IR), limiting the success of malaria control. Studies of the genetic evolution leading to insecticide resistance could enable the identification of molecular markers that can be used for IR surveillance and an improved understanding of the molecular mechanisms associated with IR. This study used a weighted gene co-expression network analysis (WGCNA) algorithm, a systems biology approach, to identify genes with similar co-expression patterns (modules) and hub genes that are potential molecular markers for insecticide resistance surveillance in Kenya and Benin. A total of 20 and 26 gene co-expression modules were identified via average linkage hierarchical clustering from *Anopheles arabiensis* and *An. gambiae,* respectively, and hub genes (highly connected genes) were identified within each module. Three specific genes stood out: serine protease, E3 ubiquitin-protein ligase, and cuticular proteins, which were top hub genes in both species and could serve as potential markers and targets for monitoring IR in these malaria vectors. In addition to the identified markers, we explored molecular mechanisms using enrichment maps that revealed a complex process involving multiple steps, from odorant binding and neuronal signaling to cellular responses, immune modulation, cellular metabolism, and gene regulation. Incorporation of these dynamics into the development of new insecticides and the tracking of insecticide resistance could improve the sustainable and cost-effective deployment of interventions.

**Supplementary Information:**

The online version contains supplementary material available at 10.1186/s12864-024-10572-z.

## Introduction

Malaria remains a significant problem in many African countries, including Benin and Kenya, where it causes a significant public health burden [1]. In Western Benin, the prevalence of malaria is exceptionally high due to favorable mosquito breeding conditions and a dense population of *Anopheles gambiae s.s.* as the main malaria vector in this region. Similarly, malaria is endemic in Kenya, with transmission occurring throughout the year, especially in the western and coastal areas [2]. The primary malaria vectors in Kenya are *An. gambiae s.l., An. arabiensis,* and *An. funestus* [3]. *Anopheles stephensi* and *An. coluzzii* have recently been reported in Northern Kenya, but their contribution to malaria transmission in Kenya is yet to be described [4, 5].

Insecticide-treated nets (ITNs) and indoor residual spraying (IRS) are key components of malaria control strategies that have been effective in reducing malaria transmission, particularly between the years 2000 and 2015 [6]. However, these measures are now threatened by insecticide resistance in mosquitoes, especially to pyrethroids. Target site mutations [7–10], over-expression of metabolic enzymes [11–18], cuticular thickening [19, 20], and changes in microbiota compositions [21, 22] have been described as mechanisms involved in conferring insecticide-resistant phenotypes. These mechanisms work synergistically to cause resistance [23].

Networks are interconnected systems of genes within an organism that interact with each other. A gene that significantly regulates other genes' activities in a network and is densely interconnected is commonly known as a hub gene [24]. Based on gene expression data, weighted gene co-expression network analysis (WGCNA) can be used to identify co-expressed modules associated with phenotypes, conditions, or traits [25]. The resulting hub genes can be identified based on the connectivity of an organism’s whole transcriptomic data. It groups genes into modules based on their expression patterns across samples and identifies hub genes that are highly connected within each module. These hub genes serve as molecular markers for the trait being studied, representing the overall characteristics of their respective modules [25]. The co-expression and similar molecular functions within modules suggest that these genes work together in response to a specific trait.

In this study, we hypothesized that genetic markers often associated with insecticide resistance are linked with additional genes in networks and that those networks are centered around hub genes. The typical differential gene expression analysis approach assumes that every gene acts as an isolated unit in the cell but does not capture gene co-expression and correlation patterns, which could provide a more holistic picture of the gene expression landscape by identifying sets of genes that regulate together to modulate the insecticide resistance phenotypes observed. WGCNA could be a beneficial approach for linking genes to insecticide-resistance phenotypes. This study applied the WGCNA approach to whole transcriptomic data to identify potential molecular markers for insecticide resistance. *Anopheles gambiae* and *An. arabiensis* samples were collected from Benin and Kenya, respectively, and the insecticide resistance phenotypes for alphacypermethrin, deltamethrin, and pirimiphos-methyl were determined in preceding studies [26, 27]. The RNA of the resistant and susceptible mosquitoes was sequenced, and the corresponding gene counts were used for WGCNA analysis to identify potential markers (hub genes) associated with insecticide resistance. After identifying the hub genes, differential gene expression analysis was performed for hub gene validation as potential IR markers.

## Results

### Data pre-processing before WGCNA

**Fig. 1:**
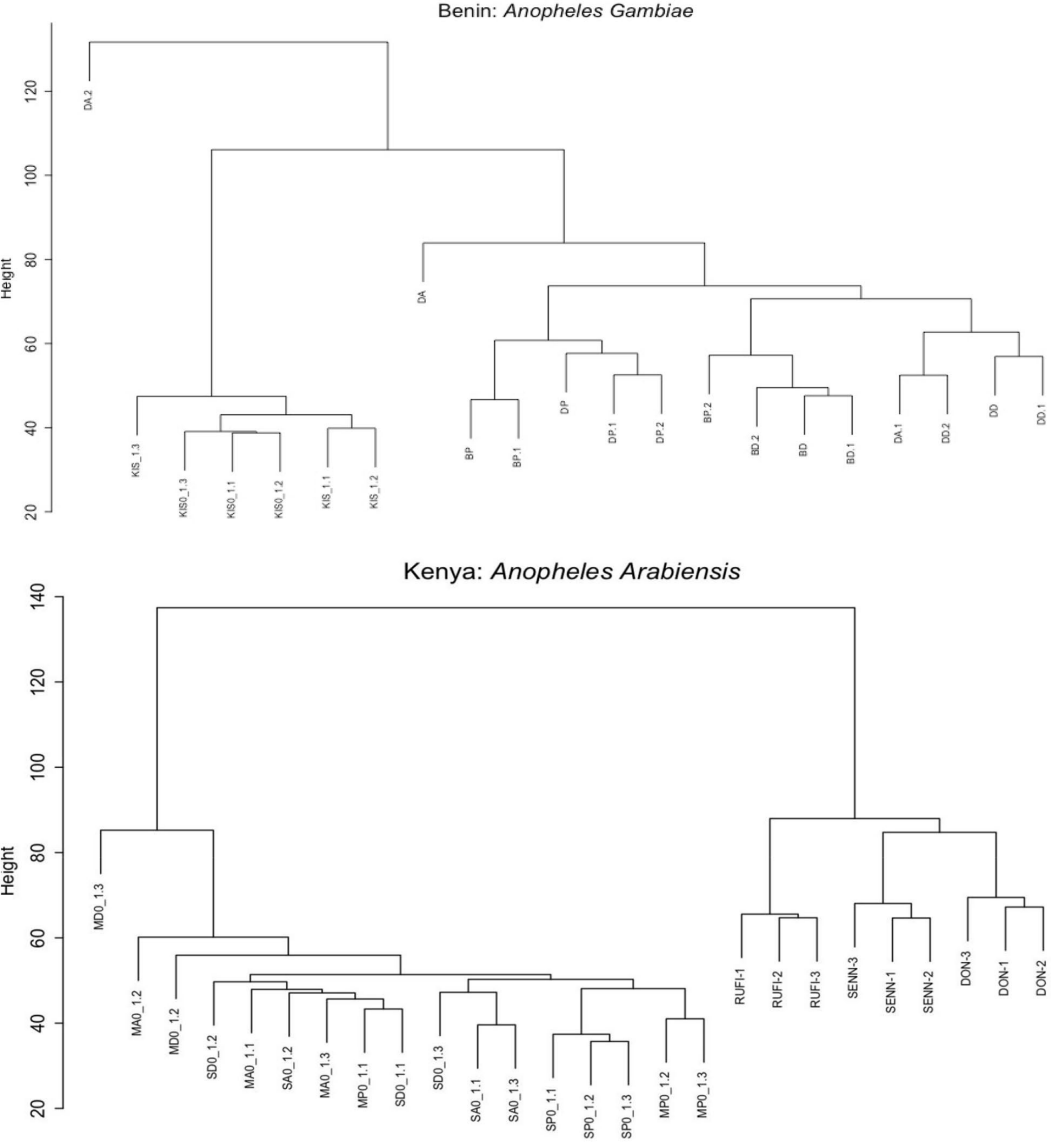
Representation plots of the total read counts for *An. gambiae* (**A**) and *An. arabiensis* (**B**). Fig 1A represents a dendrogram plot for *An. gambiae* normalized read counts. DA = Djougou Alphacypermethrin, DD = Djougou Deltamethrin, BP = Bassila Permethrin, BA = Bassila Alphacypermethrin, and BP = Bassila Primiphos-methyl. Fig. 1B represents a dendrogram plot for *An. arabiensis* normalized read counts. MA0 = Migori Alphacypermethrin, MP0 = Migori Primiphos-methyl, MD = Migori Deltamethrin, SA0 = Siaya Alphacypermethrin, SD0 = Siaya Deltamethrin, SP0 = Siaya Primiphos-methyl,KIS = Kisumu strain *An. gambiae*, and DON = Dongola strain *An. arabiensis*

The dataset initially contained a total of 17 samples from *An. gambiae* and 18 samples from *An. arabiensis*. However, we excluded two samples (DA and DA2) from *An. gambiae* and one sample (MD0_1.1) from *An. arabiensis* during the normalization process because these samples were outliers, as identified on a dendrogram plot of the total read counts at a height threshold of 80 for *An. gambiae* and 500 for *An. arabiensis* (Fig. 1).

After filtering these low abundance reads, the dataset contained a total of 10,871 and 10,908 genes from *An. gambiae* and *An. arabiensis,* respectively, which we utilized to construct weighted gene co-expression networks. This careful sample selection and noise reduction approach improved the reliability and quality of the subsequent weighted gene co-expression analysis for both *An. gambiae* and *An. arabiensis*, providing a solid foundation for the interpretation of the results in the study.

### Modules associated with insecticide resistance

**Fig. 2:**
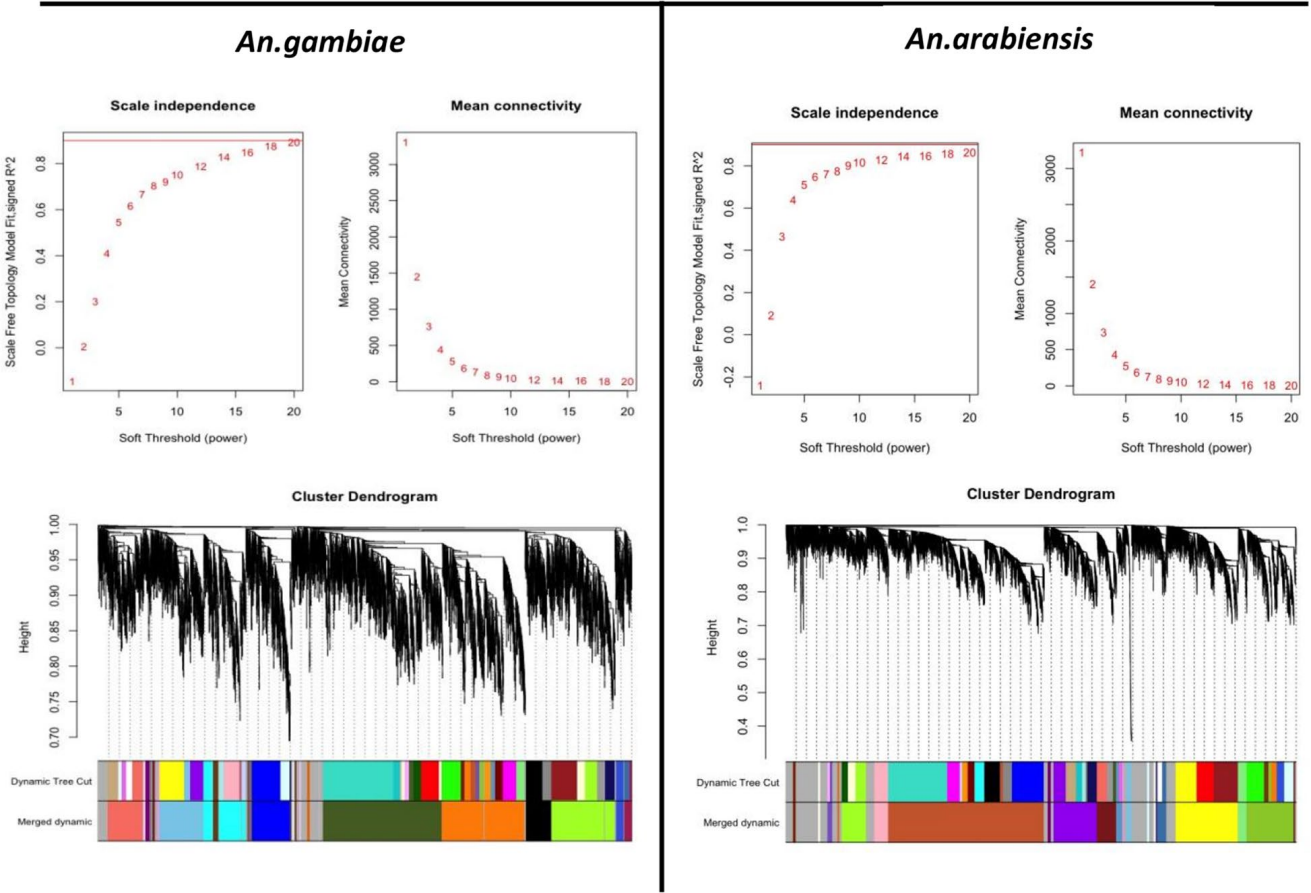
Scale-free topology fitting index (R2) versus a soft threshold power plots and hierarchical clustering dendrogram plots for both *An. gambiae* and *An. arabiensis*

**Fig. 3:**
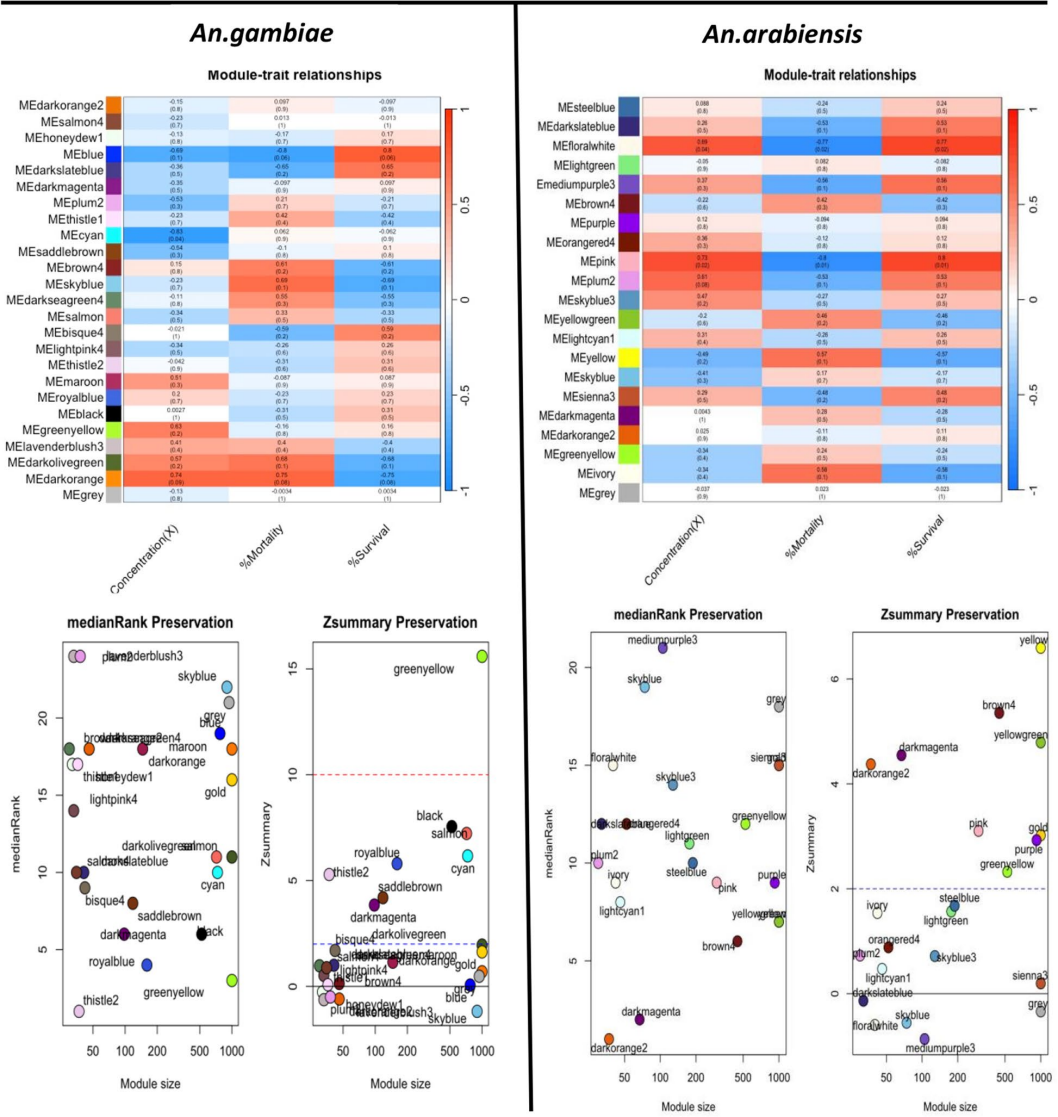
Module trait relationship heat map plots and module preservation summary plots in *An. gambiae* and *An. arabiensis*.Each point represents a module labeled by color.The dashed lines indicate thresholds Z = 2 (no preservation) and Z = 10 (highly preserved modules)

As a result, we obtained 26 gene co-expression modules for *An. gambiae* and 20 gene co-expression modules for *An. arabiensis* (Fig. 2). Module trait relationship results showed that 12 and 9 modules in *An. gambiae* and *An. arabiensis*, respectively, were positively correlated with resistance. Based on correlation with percentage survival in the bioassays, the top modules in *An. gambiae* were blue (*p* = 0.06, cor = 0.8), darkslateblue (*p* = 0.2, cor = 0.65), and bisque4 (*p* = 0.2, cor = 0.59), whereas the top three co-expression modules correlated with resistance in *An. arabiensis* were pink (*p* = 0.01, cor = 0.8), floralwhite (*p* = 0.02, cor = 0.77), and mediumpurple3 (*p* = 0.1, cor = 0.56) (Fig. 3).

**Fig. 4:**
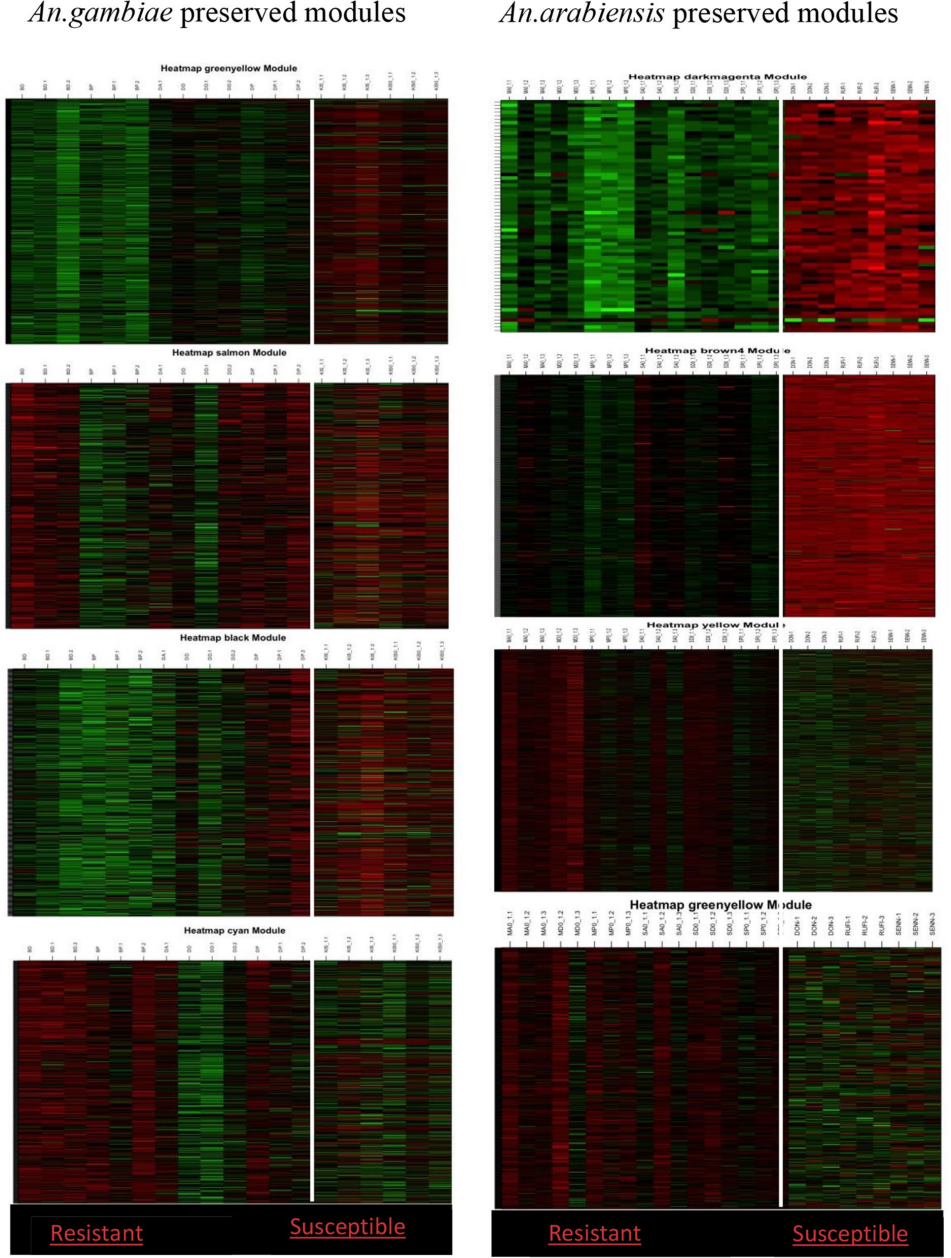
*An. gambiae* and *An. arabiensis* preserved module expression plots in resistant and susceptible anopheles populations.Higher values are represented with reds of increasing intensity, and lower values are represented with greens of increasing intensity

**Fig. 5:**
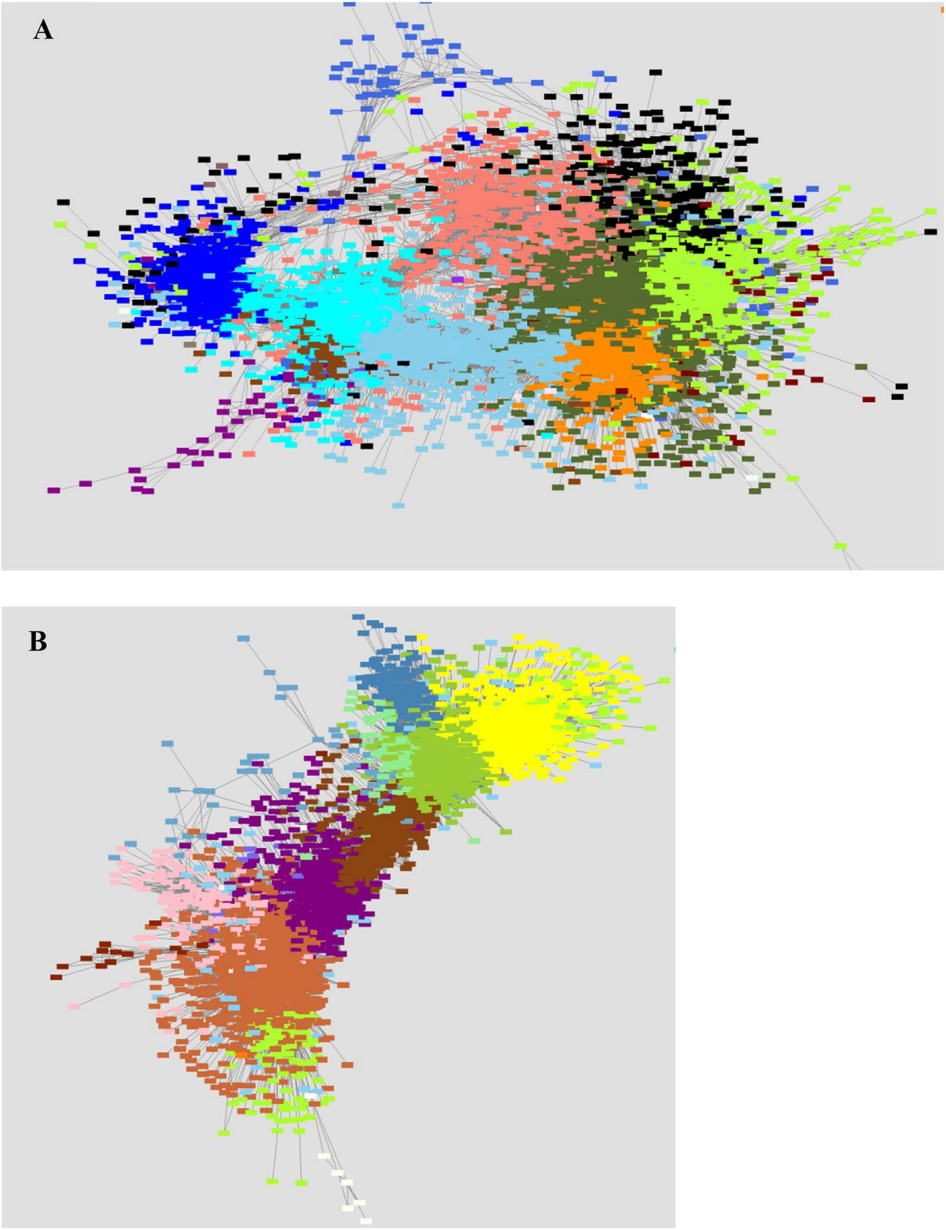
Resistant anopheles gene co-expression networks generated using weighted gene co-expression network analysis based on TOM > 0.1 for visualization. This figure illustrates gene co-expression networks; each node (point) represents a gene and genes of the same color form modules. The edges (lines) connecting the nodes represent gene-to-gene relationships. Fig. 5A represents the *An. gambiae* gene co-expression network, whereas Fig. 5B illustrates the *An. arabiensis* gene co-expression network

We then calculated module preservation in the resistant *Anopheles* network compared to the susceptible network. This analysis aided in identifying preserved modules in the resistant network and visualizing their differential expression in the two conditions. The higher the z summary score, the higher the preservation and differential expression of the two conditions. In *An. gambiae*, the top four preserved modules included greenyellow, black, salmon, and cyan (z score > 5), as shown in Fig. 3. *An. arabiensis* most preserved modules included yellow, brown, yellowgreen, and darkmagenta, which had the same z summary score as *An. gambiae* (Fig. 3). The differential expression of the top preserved modules in susceptible and resistant samples in the two species validated their role in resistance, and further investigation is recommended for the specific modules (Fig. 4). A list of all the genes in the specific modules is provided as supplementary File 2. Further analysis to explore if the number of genes in a module affected module preservation was performed using medium rank scores, and interestingly, there was no correlation between the two (Fig. 3). Further, we visualized the constructed weighted gene co-expression networks for the two species to gain insights into the relationships and interactions among various modules within the network and comprehend the interactions between genes better (Fig. 5).

### Functional enrichment of the identified modules

**Fig. 6:**
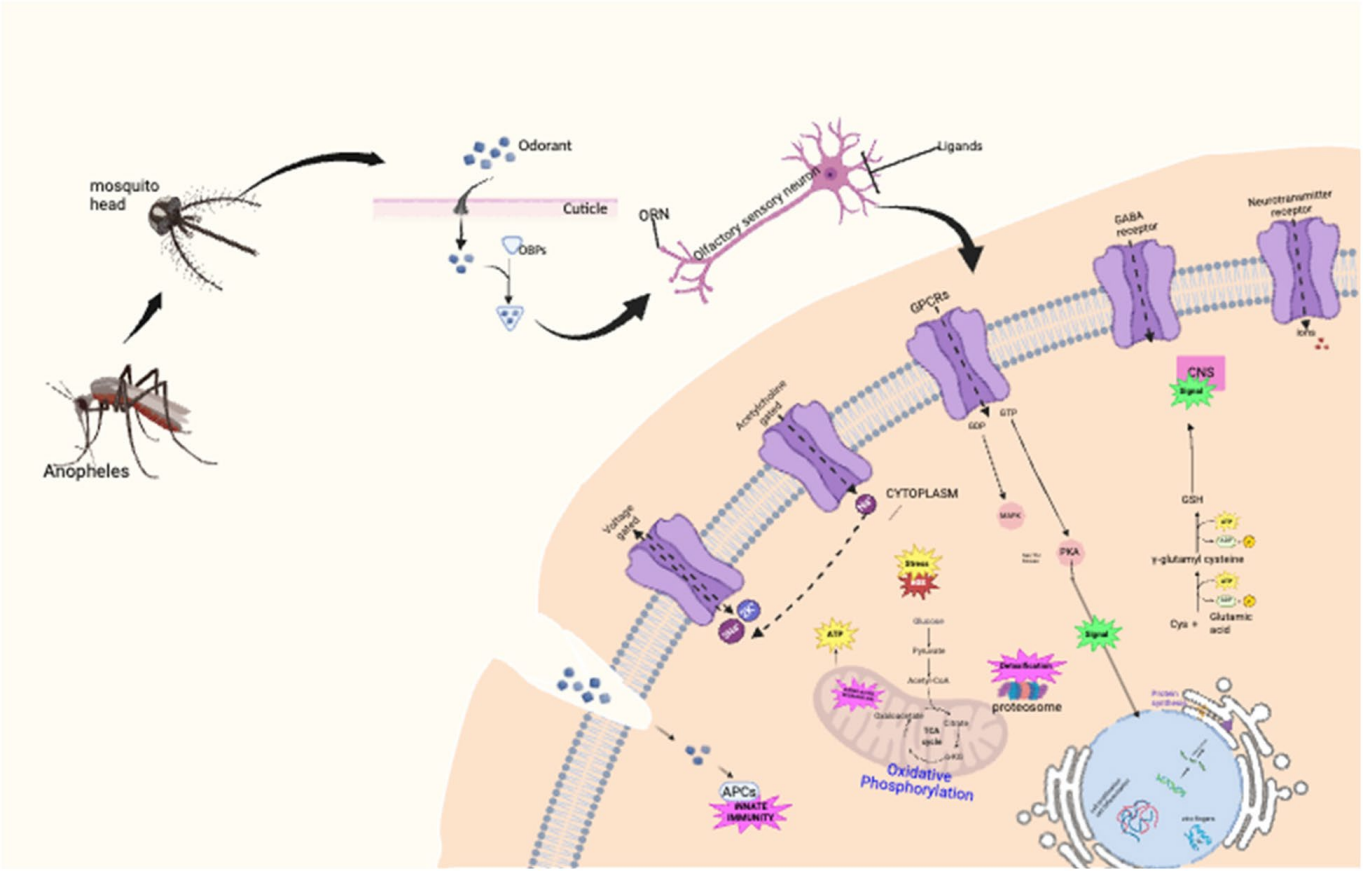
Panoramic view of molecular interactions in insecticide-resistant *Anopheles* mosquitoes. OBP-Odorant Binding Proteins; ORN-Olfactory Receptor Neurons; GPCRs-G coupled protein receptors; PKA-Protein Kinase A

To explore the functional relevance of the positively correlated modules from the module trait relationship, we performed gene ontology (GO) and Kyoto Encyclopedia of Genes and Genomes (KEGG) term enrichment analysis using the DAVID database. The enrichment analysis revealed significant enrichment of GO terms and KEGG pathways associated with genes in these modules. Enriched GO terms in all categories were analyzed. Similarly, the enriched KEGG pathways provided insights into the functional pathways involved in insecticide resistance for both *An. gambiae* and *An. arabiensis*. To visualize and explore these enrichment results, we used the EnrichmentMap plugin in Cytoscape to create an enrichment map. The enrichment map revealed multiple clusters of enriched terms for *An. gambiae* and *An. arabiensis*. The enriched terms in *An. gambiae* included transmembrane processes, immunity pathways, metabolic pathways, cytoplasm, fatty acid degradation pathways, signal transduction, and DNA replication (Supplementary 1). The enriched terms in *An*. *arabiensis* include the nucleus, metabolic pathways, signal transduction, ATP binding, and immune pathways (Supplementary 2). The enrichment map indicated several enriched insecticide targets, including voltage-gated sodium channels, acetylcholine-gated channels, and nervous system receptors. Furthermore, fundamental insecticide response mechanisms, such as immune response and metabolic pathways, were also enriched, as depicted in the maps. We constructed a detailed illustration at the cellular level that shows the molecular pathways and mechanisms in pyrethroid-resistant *Anopheles* mosquitoes using Biorender software (Fig. 6).

### Hub genes are associated with insecticide resistance

The hub genes for *An. gambiae* modules encoded 3.4 kDa salivary protein, angiotensin-converting enzyme, cuticular protein RR-2, putative serotonin 5HT-7 receptor, calcium/calmodulin-dependent serine protein kinase, ubiquinol-cytochrome c reductase subunit 8, protein disulfide isomerase family A, ceramide synthetase, cuticular protein 1 in the CPLCA family, otopetrin, zinc finger protein, CTL-like protein 2, cytosolic carboxypeptidase 6, solute carrier family 19 (thiamine transporter), RING-type domain-containing protein, E3 ubiquitin-protein ligase TRIP12, glucosylgalactosylhydroxylysine glucosidase, Cytidine deaminase, ubiquitin-conjugating enzyme E2, and 2 unspecified products (Table 1).

**Table 1:** Hub Genes and Gene Descriptions of *Anopheles gambiae* Modules. The table presents the hub genes identified from each module of *Anopheles gambiae*, along with their corresponding gene descriptions

Module	GeneID	Gene Description	Module size
Bisque4	AGAP008782	23.4 kDa salivary protein	42
Black	AGAP004563	angiotensin-converting enzyme 9	522
Blue	AGAP006369	cuticular protein RR-2 family 144	775
Brown4	AGAP004223	putative serotonin 5HT-7 receptor	46
Cyan	AGAP001683	calcium/calmodulin-dependent serine protein kinase	733
Darklateblue	AGAP006543	unspecified product	41
Darkmagenta	AGAP010337	ubiquinol-cytochrome c reductase subunit 8	98
Darkolivegreen	AGAP007393	protein disulfide isomerase family A, member 3	2452
Darkorange	AGAP001761	ceramide synthetase	1689
Darkorange2	AGAP006145	cuticular protein 1 in CPLCA family	47
Darkseagreen4	AGAP000702	otopetrin	30
Greenyellow	AGAP002705	zinc finger protein	1291
Honeydew1	AGAP010343	CTL-like protein 2	32
Lavenderblush3	AGAP001814	cytosolic carboxypeptidase 6	33
Lightpink4	AGAP006349	solute carrier family 19 (thiamine transporter), member 2/3	33
Maroon	AGAP007725	RING-type domain-containing protein	147
Plum2	AGAP004444	unspecified product	38
Royalblue	AGAP011247	Longitudinals lacking protein-like	160
Saddlebrown	AGAP013121	PH domain-containing protein	119
Salmon	AGAP001296	E3 ubiquitin-protein ligase TRIP12	717
Salmon4	AGAP008548	glucosylgalactosylhydroxylysine glucosidase	35
Skyblue	AGAP009489	Cytidine deaminase	903
Thistle1	AGAP029271	F-box domain-containing protein	36
Thistle2	AGAP005916	ubiquitin-conjugating enzyme E2 M	37

Hub genes are highly interconnected within a module and play essential roles in biological processes. The gene descriptions provide information about the function and characteristics of each hub gene

**Fig. 7:**
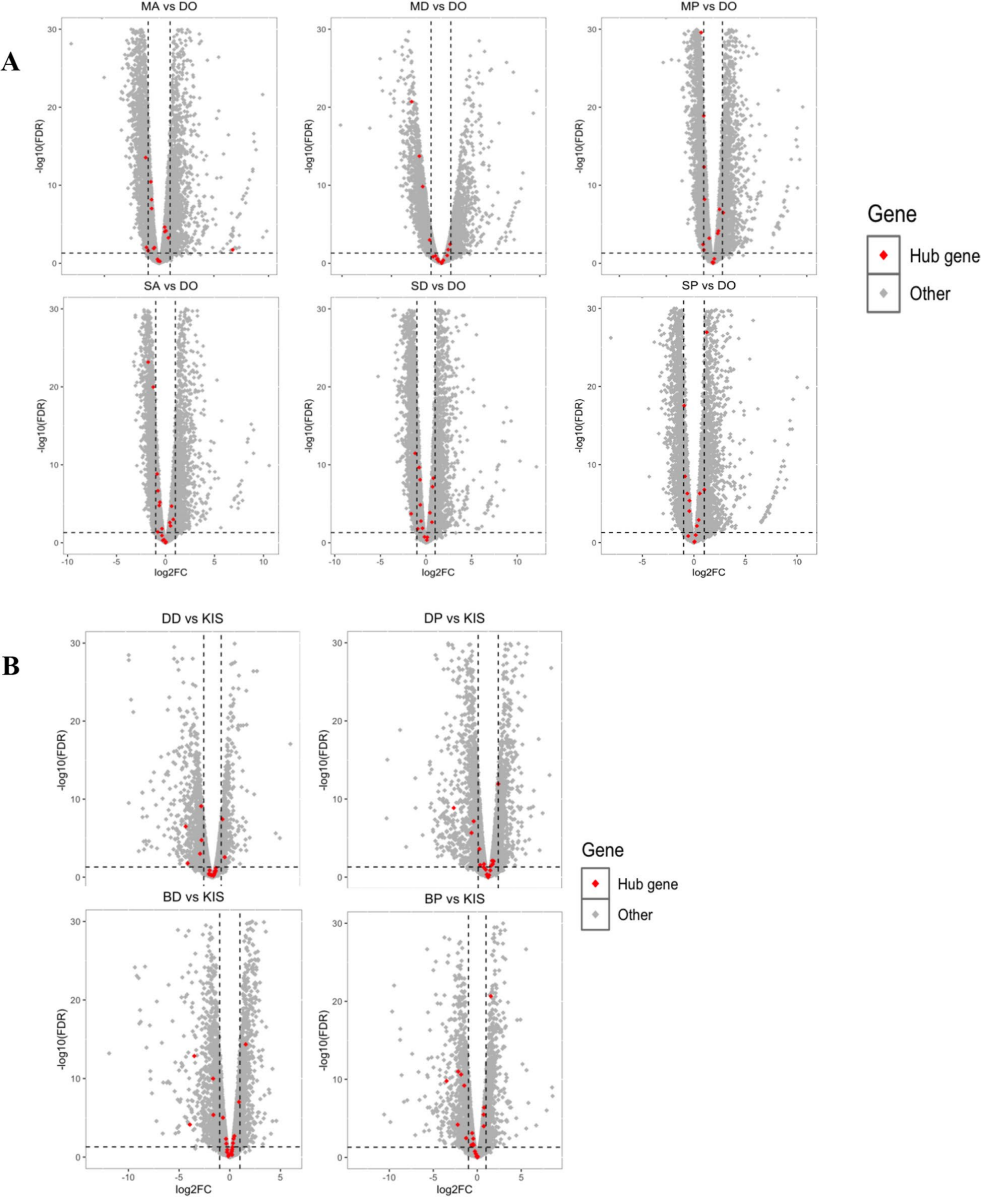
Gene expression profiles of resistant *An. arabiensis* and *An. gambiae* from (A) Kenya and (B) Benin exposed to deltamethrin, primiphos-methyl and alphacypermethrin compared to the susceptible *An. arabiensis* Dongola strain and *An. gambiae* Kisumu strain, respectively. The horizontal dotted line on the volcano plot denotes a *P*-value of 0.01, while the vertical dotted lines indicate twofold expression differences

The hub genes identified in the modules for *An. arabiensis* encode CLIP serine protease, protein chiffon, Importin subunit alpha, glutathione S-transferase zeta class, cuticular protein RR-2, E3 ubiquitin-protein ligase RNF19A, neurexin, putative muscarinic acetylcholine receptor 1, an inhibitor of apoptosis, protein wings apart-like, coronin, sortilin-related receptor, slit protein, single-stranded DNA-binding protein 3, and six unspecified products (Table 2). Notably, serine protease, E3 ubiquitin-protein ligase, cuticular protein RR2, and leucine-rich immune protein were identified as hub genes in both species. We then conducted pairwise comparisons between resistant mosquitoes in Benin and Kenya and susceptible mosquito strains to assess the differential gene expression status of the identified hub genes (Fig. 7).

**Table 2:** Hub Genes and Gene Descriptions of *Anopheles arabiensis* Modules. The table presents the hub genes identified from each module of *Anopheles arabiensis*, along with their corresponding gene descriptions

Module	GeneID	Gene Description	Module Size
Brown4	AARA000810	protein chiffon	447
Darkmagenta	AARA009424	unspecified product	67
Darkorange2	AARA003983	Importin subunit alpha	37
Darkslateblue	AARA006893	unspecified product	32
Floralwhite	AARA016743	unspecified product	40
Greenyellow	AARA015898	glutathione S-transferase zeta class	522
Ivory	AARA009171	cuticular protein RR-2 family 59	42
Lightcyan1	AARA016694	E3 ubiquitin-protein ligase RNF19A	46
Lightgreen	AARA018197	neurexin	177
Mediumpurple3	AARA006322	putative muscarinic acetylcholine receptor 1	105
Orangered4	AARA011589	unspecified product	52
Pink	AARA015714	CLIP-domain serine protease	299
Plum2	AARA007913	inhibitor of apoptosis	30
Purple	AARA010271	protein wings apart-like	922
Sienna3	AARA007630	Coronin	3328
Skyblue	AARA016867	unspecified product	95
Skyblue3	AARA018252	sortilin-related receptor	129
Steelblue	AARA007339	slit protein	189
Yellow	AARA000356	WW domain-containing protein	1330
Yellowgreen	AARA005384	single-stranded DNA-binding protein 3	1005

Hub genes are highly interconnected within a module and play essential roles in biological processes. The gene descriptions provide information about the function and characteristics of each hub gene

In *An. gambiae* populations, we conducted pairwise comparisons from two sites (Bassila and Djongou) in Benin. In Bassila, the BD vs. KIS comparison yielded 5207 differentially expressed genes, with 6 of the 24 hub genes {Cytidine deaminase (AGAP009489), Ubiquinol cytochrome c reductase (AGAP010337), Glyco_hydro_65m domain-containing protein (AGAP008548), Cuticular protein (AGAP006145), angiotensin-converting enzyme 9 (AGAP004563), and Serine/Threonine protein kinase (AGAP009784)} being significantly differentially expressed. Similarly, in the BP vs. KIS comparison, 5207 genes were differentially expressed, with the same hub genes differentially expressed in BD vs. KIS and 2 other hub genes [cuticular protein RR-2 (AGAP006369), 23.4 kDa salivary protein (AGAP008782)]. In Djougou, DD vs. KIS had 4107 DEGs, with five differentially expressed hub genes: {Cytidine deaminase (AGAP009489), Ubiquinol cytochrome c reductase (AGAP010337), Glyco_hydro_65m domain containing protein (AGAP008548), Cuticular protein (AGAP006145), and Cuticular protein (AGAP006369)}, and lastly, in DP vs. KIS comparison, we identified 5480 differentially expressed genes, with five differentially expressed hub genes: {Cuticular protein (AGAP006369), Ubiquinol cytochrome c reductase (AGAP010337), Glyco_hydro_65m domain-containing protein (AGAP008548), Ceramide synthetase (AGAP001761), and unspecified product (AGAP004444)}. In all *An. gambiae* groups, AGAP010337 and AGAP006369, encoding ubiquinol-cytochrome c reductase and cuticular protein RR-2, respectively, were significantly downregulated. Serine/Threonine Protein Kinase (AGAP009784) was upregulated in all Bassila groups.

Additionally, we did comparisons for *An. arabiensis* between two different sites: Siaya and Migori in Kenya. In the first set of comparisons, alphacypermethrin-resistant mosquitoes versus the Dongola susceptible strain (MA vs. DO), deltamethrin-resistant mosquitoes versus Dongola (MD vs. DO), and pirimiphos-methyl resistant mosquitoes versus Dongola (MP vs. DO) were compared in Migori. In the MP vs. DO comparison, we identified 6887 differentially expressed genes (DEGs) at FDR < 0.05, and among the 20 hub genes, 6 hub genes were downregulated with no upregulated hub gene. In the MA vs. DO comparison, we found 6512 DEGs, with only one unspecified protein (AARA016867) being upregulated and 6 downregulated hub genes, including protein chiffon (AARA000810), protein wings apart-like (AARA010271), Importin (AARA003983), cuticular protein RR-2 (AARA009171), and 2 unspecified products (AARA009424, AARA006322). In the MD vs. DO comparison, there were 4568 significantly differentially expressed genes; only unspecified protein (AARA006893) was upregulated, with 5 downregulated genes: {protein chiffon (AARA000810), protein wings apart-like (AARA010271), Importin (AARA003983), unspecified product (AARA009424), and cuticular protein RR-2 (AARA009171)}. Similarly, in Siaya, four hub genes {protein chiffon (AARA000810), protein wings apart-like (AARA010271), Importin (AARA003983), and unspecified product (AARA009424)} were downregulated in all Siaya comparisons, with no upregulation in SA vs. DO and SD vs. DO. The SP vs. DO comparison revealed two upregulated hub genes (serine protease (AARA015714) and unspecified protein (AARA006893)). Notably, AARA015848, encoding serine protease, and AARA001131, encoding cuticular protein, were significantly differentially expressed in most of the group comparisons.

## Discussion

This study used a systems biology method, WCGNA, to identify hub genes associated with insecticide resistance in *An. gambiae* from Benin and *An. arabiensis* from Kenya. Overall, serine protease (AARA015848), cuticular protein (AARA001131), and serine/threonine-protein kinase (AGAP009784) genes were the most upregulated hub genes. Interestingly, chitin-binding protein (AGAP008548), cuticular protein (AGAP006369), carbonic anhydrase (AGAP010337), serine/threonine protein kinase (AGAP009784), serine protease (AARA015848), and cuticular protein (AARA001131) were differentially expressed in multiple group comparisons, indicating that they play an important role in insecticide resistance and are potential molecular markers for resistant phenotypes. This analysis allows the comprehension of the “coordinative” role of the hub genes in mediating insecticide resistance, pointing these to be potentially excellent markers for monitoring insecticide resistance or targets for insecticide delivery. Further functional validation needs to be conducted to confirm their role in conferring insecticide resistance. This study provides novel insights into the differential gene expression patterns of hub genes in resistant mosquito populations, highlighting them as potential molecular markers for insecticide resistance in two main species of malaria vectors.

Understanding the genetic basis and molecular mechanisms involved in insecticide resistance is crucial for developing effective vector control strategies by identifying the most relevant targets that can be exploited to increase the efficacy of malaria control and monitor insecticide resistance development. In this study, we took a novel approach by conducting weighted gene co-expression network analysis (WGCNA) on transcriptomic data to gain insights into the connectivity of the genetic determinants and molecular processes associated with insecticide resistance in An. *gambiae* and *An. arabiensis*. To our knowledge, this is the first time a WGCNA has been applied to insecticide resistance data to identify hub genes associated with insecticide resistance.

The hub genes identified in *An. gambiae* encode ras-related and estrogen-like proteins, Cyt c reductase, cuticular protein RR-2, sodium/hydrogen exchanger, synaptotagmin-1, and leucine-rich immune proteins. These proteins have been previously implicated in various biological processes and may play essential roles in insecticide resistance. Ras-related proteins are involved in signal transduction pathways, and their dysregulation and function in oxidative stress are associated with insect insecticide resistance mechanisms [28]. Estrogen-like proteins are implicated in insect development and reproduction, and their involvement in resistance may be attributed to dysregulated hormonal signaling [29]. Cytochrome C reductase plays a vital role in the electron transport chain and the generation of reactive oxygen species during stress, potentially affecting insecticide resistance mechanisms by modulating detoxification processes [30]. Cuticular proteins form the insect cuticle, and their upregulation promotes resistance by altering insecticide penetration [31–33]. Sodium/hydrogen exchangers and synaptotagmin-1 are associated with neuronal functions and synaptic vesicle release, indicating their potential involvement in neurophysiological changes associated with resistance [34, 35]. Synaptotagmin-1 is used as a target in a yeast-interfering RNA larvicide for controlling disease vector mosquitoes, indicating that it plays a role in resistance [36]. Leucine-rich immune proteins are involved in immune response, and their upregulation may be linked to immune-related resistance mechanisms [37].

The hub genes identified in *An. arabiensis* encode CLIP serine protease, RIMS-binding protein 2, GST zeta class, slit protein, E3 ubiquitin-protein ligase RNF19B-like, cysteine desulfurase, cuticular protein RR-2, and leucine-rich melanocyte differentiation-associated protein-like. CLIP serine protease is involved in the immune response and the formation of melanin in insects [38, 39], and its role in resistance can be attributed to altered immune mechanisms. RIMS-binding protein 2 is involved in neurotransmitter release and synaptic function [40], implying that it plays a potential role in neurophysiological changes associated with resistance. Glutathione S transferases, class Zeta, are part of the GST family, and several studies have demonstrated their role not only in insecticide detoxification processes but also in plasmodium infection in *Anopheles* and *Aedes* species [13, 41–44]. Slit protein is involved in axon guidance and may play a role in resistance-related neuronal adaptations. E3 ubiquitin-protein ligase RNF19B-like is involved in protein degradation pathways and may modulate the turnover of proteins associated with resistance mechanisms [45]. Transcriptomic analysis has shown that cuticular proteins and salivary gland proteins are implicated in insecticide resistance [26, 27, 46, 47]. Notably, we identified four hub genes, namely serine protease, E3 ubiquitin-protein ligase, cuticular protein RR2, and leucine-rich immune protein, shared between the two resistant *Anopheles* species, indicating the possibility of common resistance mechanisms across the two species.

The identified preserved modules in both *An. gambiae* and *An. arabiensis*, showing differential expression between susceptible and resistant samples, highlight the significant role of these modules in resistance. This emphasizes the need for deeper investigation into the specific genes within these modules to understand their functional contributions to resistance mechanisms. The hub genes within these preserved modules included zinc finger proteins, E3 ubiquitin protein ligase, angiotensin, calcium-dependent serine protein kinase, protein shifon, glutathione S transferase, and unspecified protein. Further, KEGG and GO analyses were conducted to provide insights into the functional relevance of these genes and molecular mechanisms associated with insecticide resistance in resistant *Anopheles*, as illustrated in Supplementary File 1. This integration of enrichment analysis with network analysis provided a comprehensive overview of the biological processes, cell organelles, and pathways associated with insecticide resistance in *An. gambiae* and *An. arabiensis*.

Enriched GO terms related to transmembrane processes indicate the importance of membrane transporters and channels in these resistant a*nopheles*, especially with their role as pyrethroid targets [48]. The enrichment of immune response-related terms highlighted the involvement of immune pathways in resistant mosquitoes, indicating the activation of immune defense mechanisms in resistant species. Upregulation of immune genes in field mosquitoes due to exposure to different environmental bacteria, parasites, and viruses. To reduce this environmental effect on gene expression, these samples were reared in the laboratory to F0 generation and then exposed to insecticides. Therefore, we are still convinced that their overrepresentation in the network may be due to the insecticide exposure. Metabolic pathways were also enriched in the analyzed dataset, implying the potential role of metabolic adaptations in detoxification and resistance. The top enriched pathways included fatty acid degradation, signal transduction, DNA replication, ATP binding, and oxidative phosphorylation. This enrichment analysis, in conjunction with the existing literature, enabled us to get a step-by-step molecular mechanism of resistance at a cellular level, as illustrated in Fig. 5.

The intake of insecticides by mosquitoes involves several interconnected processes. First, odorants present in the environment permeate the mosquito cuticle and bind to Odorant Binding Proteins (OBPs). Odorant Receptor Neurons (ORNs) within the mosquito's sensory system recognize the bound odorants and trigger specific cellular responses [49]. These responses involve the activation of various receptors, including GPCRs, GABA receptors, and ion channels on the cell membrane, leading to activating signaling pathways. For example, the GABA receptor pathway involves the production of gamma-aminobutyric acid (GABA) and its subsequent signaling to the central nervous system [50]. Cellular gates and receptors facilitate the movement of ions in and out of the cell, regulating neuronal activity. The proteasome plays a significant role in insect resistance to insecticides by facilitating the breakdown and removal of insecticide-targeted proteins, enabling some insects to develop resistance mechanisms. Insects with increased proteasome activity can more efficiently degrade or detoxify insecticides, reducing their toxic effects and contributing to the development of insecticide resistance [51]. Mitochondria play a vital role in cellular energy production through the tricarboxylic acid (TCA) cycle and oxidative phosphorylation. The mosquito immune response can also be influenced by odorants, which can be captured by antigen-presenting cells (APCs) to induce an immune reaction. In summary, the intake of insecticides by mosquitoes involves a complex series of steps, including odorant binding, neuronal signaling, cellular responses, immune modulation, cellular metabolism, and gene regulation, which are crucial in the activity or detoxification of insecticides. These findings can provide a reference for developing new insecticides with higher efficacy and specificity and monitoring the emergence of resistance via key biological targets.

## Conclusion

We performed weighted gene co-expression network analysis (WGCNA) and differential expression analysis to unravel the genetic determinants and molecular processes driving insecticide resistance in *An. gambiae* and *An. arabiensis*, providing key insights into the multifactorial nature of insecticide resistance. The identified hub genes can be used to understand the mechanisms that could be targeted to improve mosquito control. These genes are implicated in various biological functions, including signaling pathways and oxidative stress responses to immune defenses and nervous system adaptations, showing the complexity of the development of insecticide resistance at the molecular level. The discovery of shared hub genes between the two *Anopheles* species indicates that they may share some common mechanisms for resistance and can be used to develop strategies targeting both species. These findings provide a novel basis for analyzing and interpreting transcriptomic data associated with insecticide resistance.

## Methods

### Data acquisition

We retrieved RNA-Seq data on insecticide-resistant *An. gambiae s.s.* and *An. arabiensis* from the NCBI repository (SRA PRJNA986474 and PRJNA98270). The Genomics of African Vectors for NMCP Management of Insecticide Resistance (G-AVENIR) project generated the two datasets that aimed to identify molecular markers associated with insecticide resistance. The first dataset consisted of 17 resistant samples of *An. gambiae* collected from Benin {Djougou (9° 42′ 29.1312″ N and 1° 39′ 58.8672″ E) and Bassila (9° 0′ 23.0148″ N and 1° 39′ 50.1264″ E)} collected in August and October 2019 respectively, with each sample comprising a pool of 10 mosquitoes. These mosquitoes were phenotyped for resistance using CDC bottle bioassays to different insecticides (alphacypermethrin, deltamethrin, and pirimiphos methyl [52]. The second dataset consisted of 18 resistant samples of *An. arabiensis* collected from Kenya {Migori (1.0707° S, 34.4753° E) and Siaya (0.0626° N, 34.2878° E)} in August and October 2019, respectively, with each sample also comprising a pool of 10 mosquitoes. The mosquitoes in the second dataset were also phenotyped for resistance to the same insecticides as the Benin samples [53].The CDC bottle bioassay, sample processing, RNA extraction, and library preparation of the data utilized in this study are described in [26, 27]. We also added susceptible laboratory population data from the *An. gambiae* Kisumu strain and *An. arabiensis* Dongola datasets (SRA PRJNA986474 and PRJNA98270) for WGCNA and differential gene expression analysis.

### Transcriptomic data processing and filtering

We initiated the RNA Pipeline as used by [52, 53] to assess the quality of the RNA-Seq raw reads using the FastQC (v0.11.5, [54]) software. To ensure the reliability of our data, we then used fastp (-l50,-q20,v0.20.1, [55]) software to eliminate low-quality reads and adaptor sequences. We then aligned the trimmed sequences to their respective reference genomes (*An. gambiae* (release 48), *An. arabiensis* Dongola (release 57)) from the Vectorbase database [56] using subjunc (v1.6.0, [57]). Subsequently, we subjected the resulting BAM files to post-alignment processing steps using samtools (v1.17, [58]) to remove duplicates, low mapping quality reads (-q 10), and unmapped reads (-F4) to have accurate and reliable results for downstream analysis. Next, we quantified the filtered and sorted mapped reads using FeatureCounts (v1.6.0, [59]) software. We excluded read counts that were below 10 and were present in over 80% of the dataset to minimize noise during correlation analysis. This step was essential for maintaining the robustness of our downstream analysis. We were interested in coding genes and their functional analysis, so we eliminated non-coding genes from the annotated gene count list.

### Read count normalization

We normalized the *An. gambiae* and *An. arabiensis* read counts separately because they were from distinct investigations. We used the *CalcNormFactors* function in EdgeR (v3.14.0) [60] to compute normalization factors using the Trimmed Mean of M-Values (TMM) normalization method. After normalization, we constructed a dendrogram plot for the counts using hclust in R (v1.2.3) to identify outliers based on the distance matrix [61]. Only samples below cut heights of 500 and 80 for *An. arabiensis* and *An. gambiae*, respectively, were used for the WGCNA.

### Construction of gene co-expression network

The normalized counts were utilized to construct a weighted gene co-expression network using the WGCNA R package version 1.72–1 (Langfelder & Horvath, [25]). We used the *pickSoftThreshold* function to determine the soft-thresholding power (β) and created a weighted adjacency matrix for a signed network type based on the β using the *adjacency* function. The matrix was then transformed into the Topological Overlap Matrix (TOM) using the *TOM similarity* function, and the TOM measure between gene pairs was utilized for average linkage hierarchical clustering (soft-power 18/20, mergeCutheight 0.25, minModuleSize 30, networktype signed). We then calculated the module-trait relationships by evaluating Pearson’s correlation between the eigengene of each module and the specific phenotype data (concentration of the insecticides, percent mortality, and percent survival, Supplementary 3) for the samples. Module eigengenes were calculated separately in each network using the *moduleEigengenes* functions in WGCNA. Module preservation statistics were calculated using the *modulePreservation* function in the WGCNA R package (nPermutations = 100, random seed = 1) by comparing the resistant sample network with the susceptible network [62]. Lastly, we identified the hub genes using the *chooseTopHubInEachModule* function (with a power of 4 and signed type) in WGCNA.

### Differential gene expression analysis

To evaluate the differential expression status of the identified hub genes within the *An. arabiensis* and *An. gambiae* networks, we conducted a comprehensive differential gene expression analysis. This analysis involved a direct comparison between the expression levels of these hub genes in the resistant population (survivors after insecticide exposure) versus the susceptible population (Kisumu strain and Dongola for *An. gambiae* and *An. arabiensis,* respectively). We statistically assessed differential expression using the *Likelihood-Ratios (LR) test* implemented in edgeR. Genes were classified as differentially expressed if they exhibited a significant change in expression with a false discovery rate (FDR)-adjusted *p*-value less than 0.05, indicating a high confidence level in the results. Our particular focus was on hub genes that demonstrated a fold change exceeding two and satisfied the strict FDR criteria, as these genes were the most likely potential insecticide resistance markers.

### Functional enrichment analysis and visualization

We conducted GO and KEGG pathway analysis using the Database for Annotation, Visualization, and Integrated Discovery (DAVID) database [63] to understand the biological functions of the modules. We utilized Cytoscape to visualize the weighted gene co-expression networks for *An. gambiae* and *An. arabiensis* with default settings. An enrichment map, a Cytoscape [64] plug-in, was used to identify the enriched terms in a network. The molecular pathways associated with insecticide resistance phenotypes were illustrated using Bio-Render software [65].

### Supplementary Information

Supplementary Material 1.

Supplementary Material 2.

Supplementary Material 3.

## Data Availability

Sequence data used by this study is available at Sequence Read Archive (SRA) under the Bio Project accession number  PRJNA986474 and PRJNA98270. Custom scripts used for all the analysis are available from the primary author on request.Email:cynthiaawuor18@gmail.com.
