# Supplementary material for: Key gene modules and hub genes associated with pyrethroid and organophosphate resistance in Anopheles mosquitoes: a systems biology approach
Source: BMC Genomics. 2024 Jul 3;25:665. doi: 10.1186/s12864-024-10572-z (PMC11223346; doi:10.1186/s12864-024-10572-z)
Supplement: Supplementary file 1 — Supplementary Material 1. [file 12864_2024_10572_MOESM1_ESM.docx]

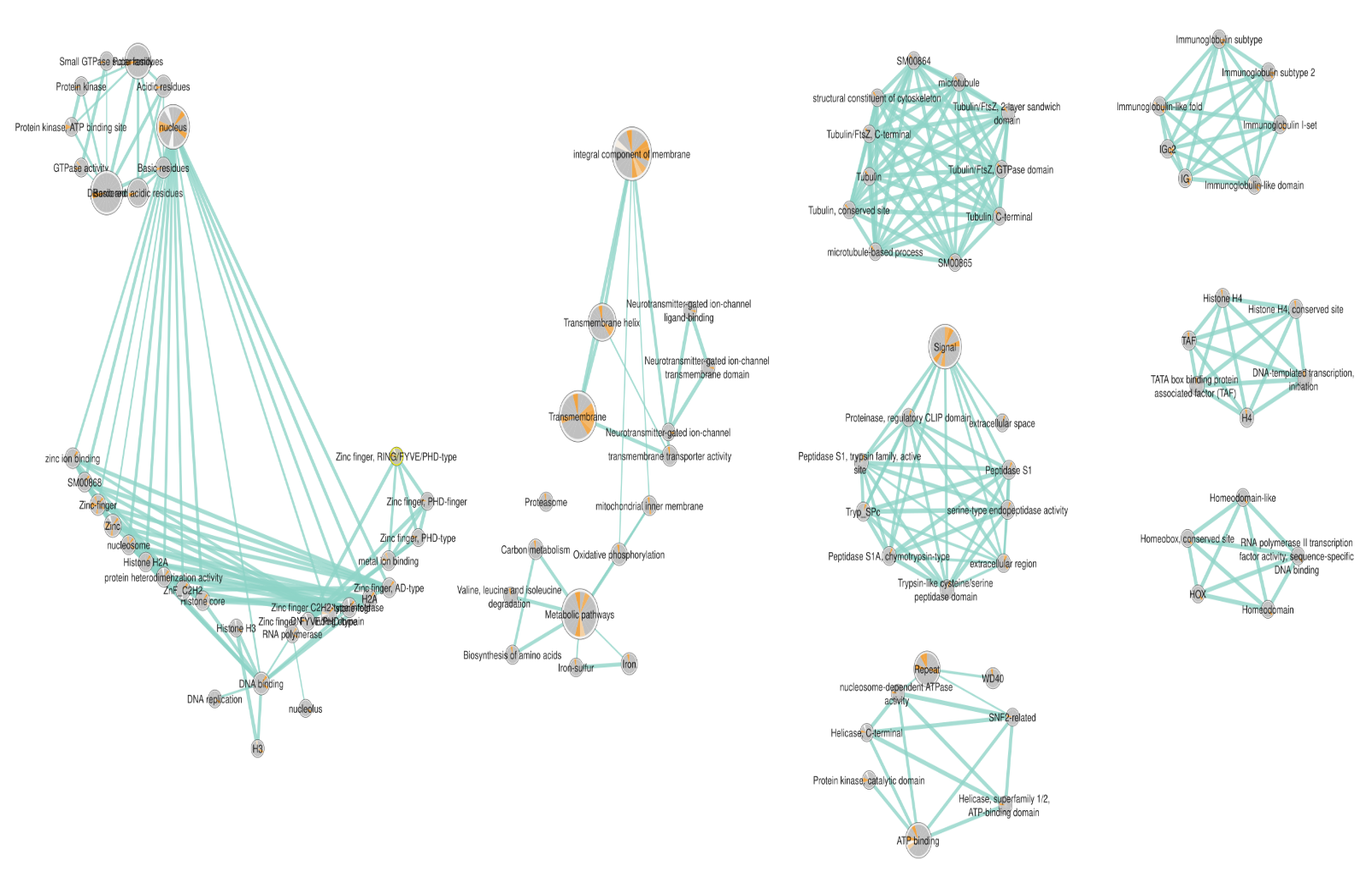


**Supplementary 1**

Illustration of an Enrichment Map for *Anopheles gambiae* Coexpression Network. A visual representation of the *Anopheles gambiae* coexpression network's enriched terms, encompassing pathways, Gene Ontology (GO), and Kyoto Encyclopedia of Genes and Genomes (KEGG) terms.


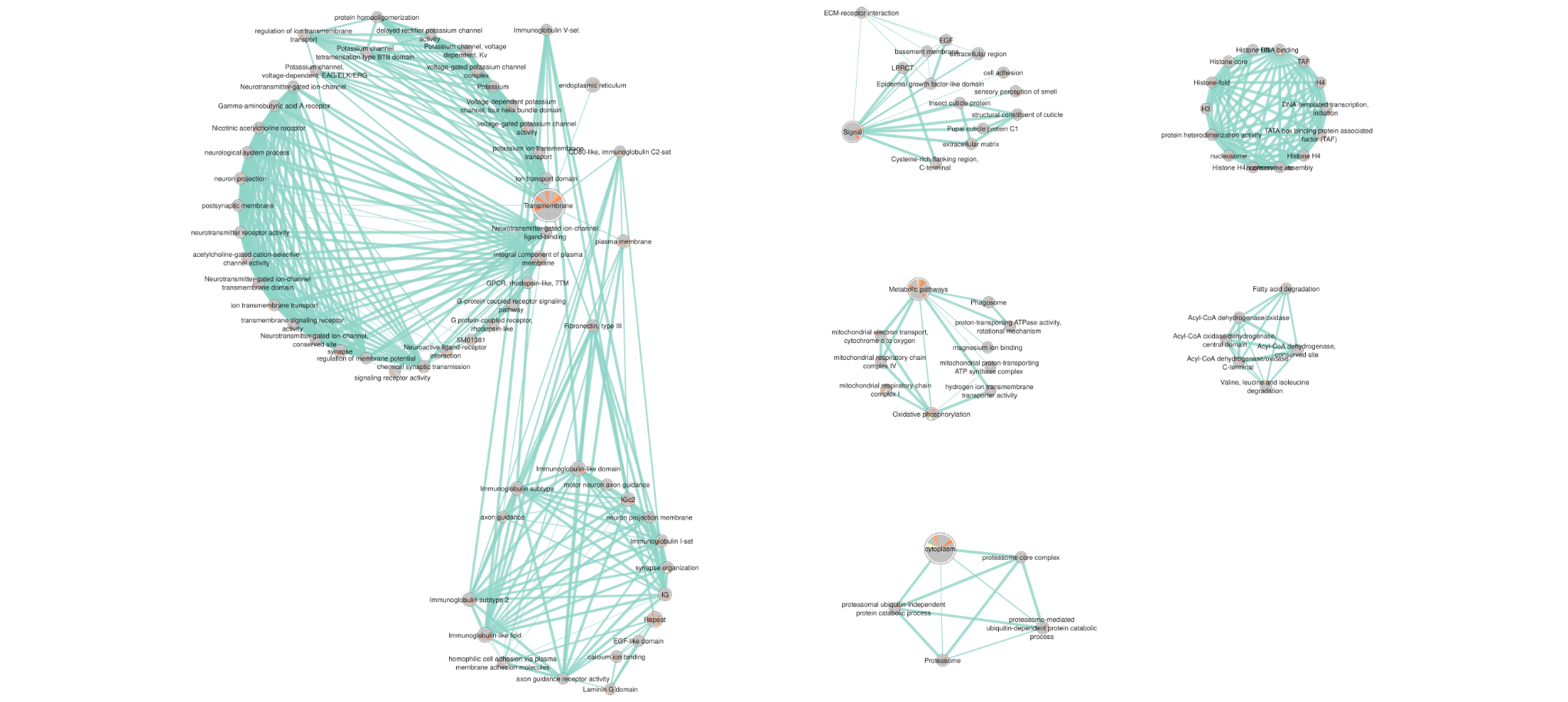


**Supplementary 2**

Illustration of an Enrichment Map for *Anopheles arabiensis* Coexpression Network. A visual representation of the *Anopheles arabiensis* coexpression network's enriched terms, encompassing pathways, Gene Ontology (GO), and Kyoto Encyclopedia of Genes and Genomes (KEGG) terms.
